# Physiological Incest

**Published:** 1860-07

**Authors:** 


☞ In the April number of the *Journal*, we published, as original, an article under the caption of "Physiological Incest," which appeared also the same month as an original communication to the *Louisville Monthly Medical News*, and in the May number of the *Charleston Medical Journal and Review*. We knew nothing, at the time, of the author of the article, and the sole merit of the paper—its brevity—coupled with a degree of inadvertancy on our part, gave it a place in the pages of the *Journal*. The simultaneous furnishing of the same article to different medical publications, is not recognized as honorable by any journalists, and can only be looked upon as a disreputable expedient to bring the author's name before the public; and consequently we were prepared and gratified to learn that the writer was not a recognized member of the medical profession being only one of the legion of pretenders that hover about it, as the mercenary camp followers do about an army, for purposes of plunder. We do not feel ourselves in any way censurable for the occurrence, yet we deem this explanation proper to be made.

